# Effect of Ultrasonic Vibration on Microstructure and Fluidity of Aluminum Alloy

**DOI:** 10.3390/ma16114110

**Published:** 2023-05-31

**Authors:** An Li, Zhiming Wang, Zhiping Sun

**Affiliations:** School of Mechanical Engineering, Qilu University of Technology (Shandong Academy of Sciences), Jinan 250353, China

**Keywords:** ultrasonic casting, aluminum alloy, fluidity, fluid simulation, dendrite growth

## Abstract

The effect of ultrasonic vibration on the fluidity and microstructure of cast aluminum alloys (AlSi9 and AlSi18 alloys) with different solidification characteristics was investigated. The results show that ultrasonic vibration can affect the fluidity of alloys in both solidification and hydrodynamics aspects. For AlSi18 alloy without dendrite growing solidification characteristics, the microstructure is almost not influenced by ultrasonic vibration, and the influence of ultrasonic vibration on its fluidity is mainly in hydrodynamics aspects. That is, appropriate ultrasonic vibration can improve fluidity by reducing the flow resistance of the melt, but when the vibration intensity is high enough to induce turbulence in the melt, the turbulence will increase the flow resistance greatly and decrease fluidity. However, for AlSi9 alloy, which obviously has dendrite growing solidification characteristics, ultrasonic vibration can influence solidification by breaking the growing α (Al) dendrite, consequently refining the solidification microstructure. Ultrasonic vibration could then improve the fluidity of AlSi9 alloy not only from the hydrodynamics aspect but also by breaking the dendrite network in the mushy zone to decrease flow resistance.

## 1. Introduction

The demand for lightweight materials in the machinery industry has led to the widespread use of aluminum alloys. However, as the structure of aluminum castings becomes increasingly complex to meet application requirements, the production of high-quality castings faces significant challenges. To address this, many efforts have been made to improve traditional casting technology. One promising method is ultrasonic-assisted casting, which can refine the grain size of alloys and improve the quality and mechanical properties of castings [1,2].

In the past, most of the ultrasonic-assisted casting process was in the form of directly inserting an ultrasonic horn into the melt [3,4,5,6]. However, due to the attenuation of ultrasonic energy, this method is not suitable for castings with complex structures and is more commonly applied in semi-continuous casting processes [7,8,9]. Aiming to overcome this disadvantage, attempts have been made in recent years to use an ultrasonic horn as part of the mold cavity. For example, Mukkollu et al. applied ultrasonic vibration to stainless steel molds and combined ultrasonic with slope casting to obtain ingots with finer microstructures and higher performance [10]. Peng Yin et al. used a steel clamp to fix sand molds and applied ultrasonic vibration to the resin sand mold through the clamp to refine the microstructure of aluminum casting [11]. If elaborately schemed, ultrasonic vibration applied to molds theoretically can be suitable for any complex casting, which is an important direction in realizing the application of ultrasonic vibration to complex shape castings. For castings with complex shapes and structures, the casting quality not only involves microstructure determined by solidification but also depends greatly on the casting ability related to flow, mold filling, and shrinkage [12,13,14]. However, previous studies on ultrasonic-assisted casting mainly focused on its role in refining microstructure and rarely involved the effects regarding the flow and filling properties of the casting.

It has been found that wall vibration can change the velocity field of the fluid near the wall and affect the flow characteristics of the fluid [15,16,17,18]. However, excessive violent vibration can excite turbulence and consequently increase flow resistance significantly [19,20]. Ultrasonic vibration, a type of mechanical wave with a frequency exceeding 20 KHz, inevitably affects the flow field changes during the filling process of the melt. In addition, unlike pure fluids, the flow of melt is also significantly influenced by solidification factors [21,22]. In particular, ultrasonic vibration can break dendrites during solidification [23,24]. Although there are few reports, this dendrite fragmentation effect can theoretically also promote the fluidity of castings [25]. Therefore, changes in alloy fluidity under ultrasonic vibration are influenced by both solidification and hydrodynamics factors. However, there is currently limited research in this area, especially regarding the relationship between the two factors, which is not yet clear.

In order to promote the application of ultrasonic vibration in improving alloy fluidity and enhancing casting quality, in the present work, uniform ultrasonic vibration of mold walls was achieved through resonance. Based on this, the effect of ultrasonic vibration on the fluidity and solidification structure of cast aluminum alloys (AlSi9 and AlSi18 alloys) with different solidification characteristics was investigated. Furthermore, the impact of ultrasonic vibration on the flow field during the molten metal filling process was analyzed using fluid simulation software (ANSYS-FLUENT). Based on solidification structure analysis and fluid simulation, the mechanism by which ultrasonic vibration affects the fluidity of cast aluminum alloys was studied from two perspectives: the impact on dendritic growth during solidification and hydrodynamic factors.

## 2. Experimental Procedure

Binary hypoeutectic AlSi9 and hypereutectic AlSi18 alloys were selected to investigate the influence of ultrasonic vibration on their fluidity in the casting process. The alloys were prepared from pure aluminum (99.9 wt.%) and Al-20 wt.% with Si master alloys according to the nominal composition.

**Figure 1:**
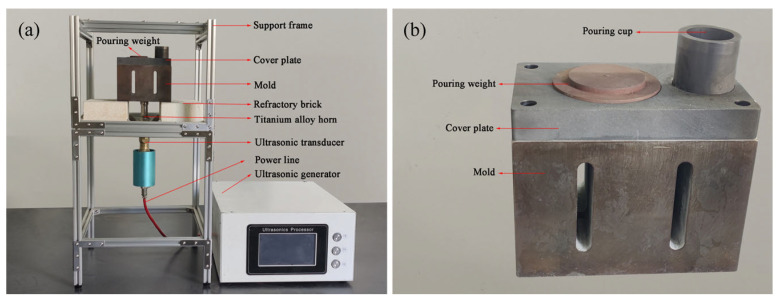
Ultrasonic vibration system: (**a**) experimental setup; (**b**) metal mold.

The ultrasonic vibration system used in the present work consisted of a support ultrasonic generator (TL-1200 × 3, infinitely adjustable power 0–1200 W), a 24,000 Hz ultrasonic transducer, a Ti6Al4V horn, and a metal mold (4Cr5MoSiV1), as shown in Figure 1.

**Figure 2:**
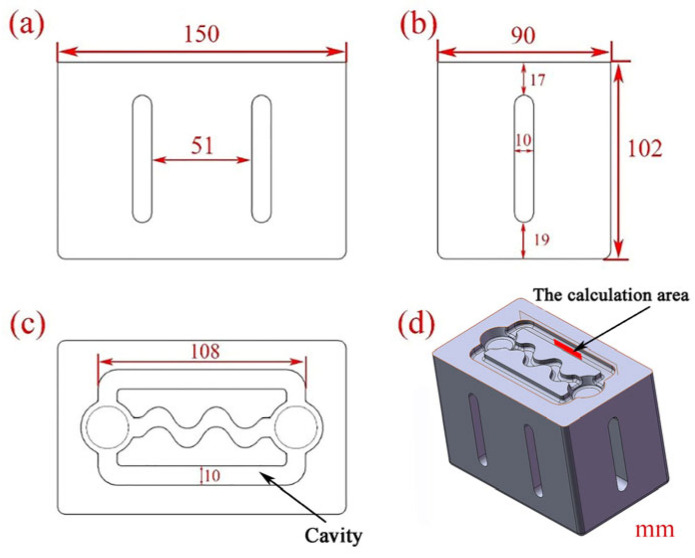
Fluidity test mold: (**a**) main view; (**b**) left view; (**c**) top view; (**d**) isometric view.

To ensure efficient vibration of the mold cavity under resonance mode, the mold’s overall structure was specially designed based on modal analysis conducted using COMSOL Multiphysics software, as illustrated in Figure 2.

**Figure 3:**
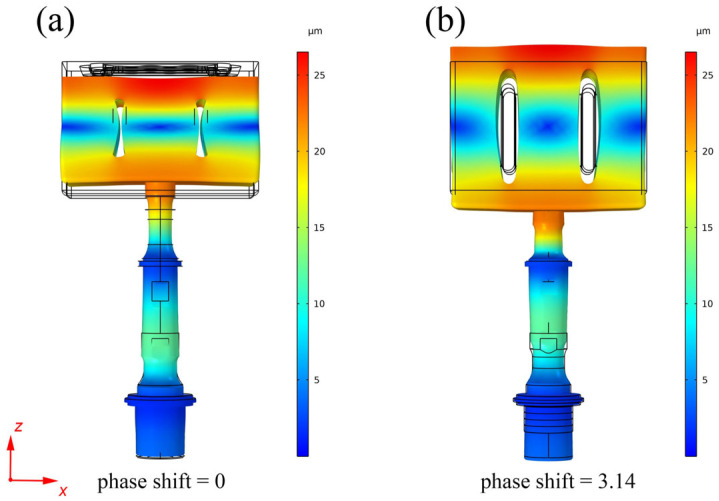
Simulated resonance vibration of the fluidity test mold in the half vibration cycle: (**a**) phase shift 0; (**b**) phase shift 3.14.

Figure 3 displays the vibration modal shape of the mold in the half cycle (phase shift 0–3.14) under ultrasonic excitation, as simulated using COMSOL Multiphysics software. The material properties utilized for the simulation are listed in Table 1. The results reveal that the mold vibration takes the form of elastic volume deformation, primarily along the Z axis perpendicular to the bottom of the cavity.

**Figure 4:**
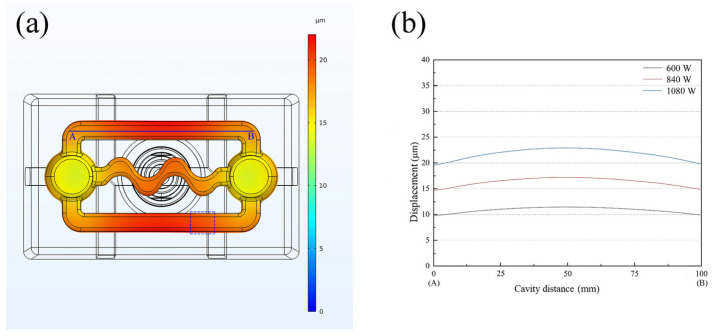
Vibration of mold cavity under different ultrasonic powers: (**a**) vibration mode of the mold cavity; (**b**) distribution of vibration amplitude along the blue line segment A–B in subfigure (**a**).

Vibration of the mold cavity is the primary factor influencing the fluidity of the melt. Consequently, the displacement of the mold cavity surface was extracted and is depicted in Figure 4. The results indicate that the surface displacement of the mold cavity was relatively uniform and increased gradually with the rise in input power. The maximum surface displacement of the mold cavity (which represents the vibration intensity) can reach approximately 20 μm with an input ultrasonic power of 1080 W. The upper cover plate, which was integrated with the sprue and riser of the mold, was constructed of graphite and was not rigidly connected to the mold to avoid influencing the vibration mode, as depicted in Figure 1b.

Aluminum alloys AlSi9 and AlSi18 were melted using a crucible resistance furnace (SG2-5-10) and subjected to slag removal and degassing before being held at 973 K and 1003 K, respectively. Prior to casting, the mold must be preheated to 473 K, connected to the ultrasonic horn, and then positioned on the bracket. The level of the mold was adjusted using a level gauge, and ultrasonic vibration was activated while pouring the aluminum alloy melt into the mold to evaluate its liquidity. After pouring, the ultrasonic vibration was turned off until solidification was complete. To assess fluidity, the cavity of the bending part in the middle of the mold was obstructed, allowing the melt to flow only through the straight sections on both sides, as shown in Figure 2. The length of the two straight sections of the fluidity sample was measured, and the average value was calculated to determine its fluidity.

To explore the impact of ultrasonic power on the fluidity and microstructure of alloys with distinct solidification modes, binary hypoeutectic AlSi9 and hypereutectic AlSi18 were chosen as the experimental materials, considering their different solidification temperature ranges. To this end, a comparative experimental plan was developed, as illustrated in Table 2.

The analysis of ultrasonic vibration on the solidification structure was carried out on the designated position of the casting samples marked by the blue dotted box in Figure 4. After polishing, the samples were etched using Keller’s reagent (2.5% HNO_3_ + 1.5% HCL + 1% HF + 95% H_2_O), and their microstructures were analyzed using Leica DM2700M microscopy (Leica, Wetzlar, Germany) and Oxford Nordy Max3 electric backscatter diffusion (EBSD) (Oxford Instruments, Abingdon, UK).

## 3. Results and Discussion

### 3.1. Effect of Ultrasonic Power on Alloy Fluidity

**Figure 5:**
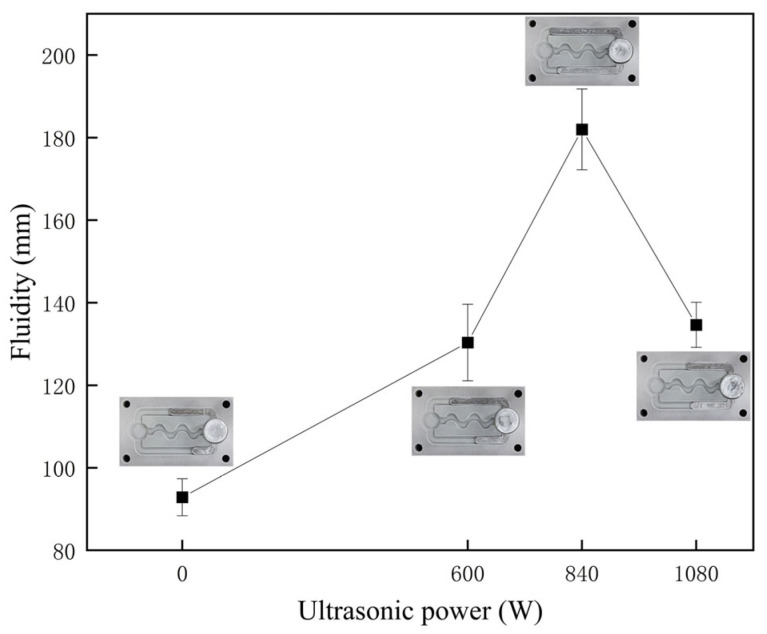
The influence of ultrasonic power on the fluidity of AlSi9 alloy.

The impact of ultrasonic power on the fluidity of AlSi9 alloy is depicted in Figure 5. The results indicate that the fluidity of the alloy increased initially with an increase in ultrasonic power until it reached a peak and then decreased. The flow length of AlSi9 alloy substantially improved from 92.9 ± 4.5 mm to 182.0 ± 9.8 mm by applying 840 W ultrasonic vibration. However, upon further increasing the ultrasonic power to 1080 W, the flow length of the alloy started to decline, akin to the effect induced by 600 W power.

**Figure 6:**
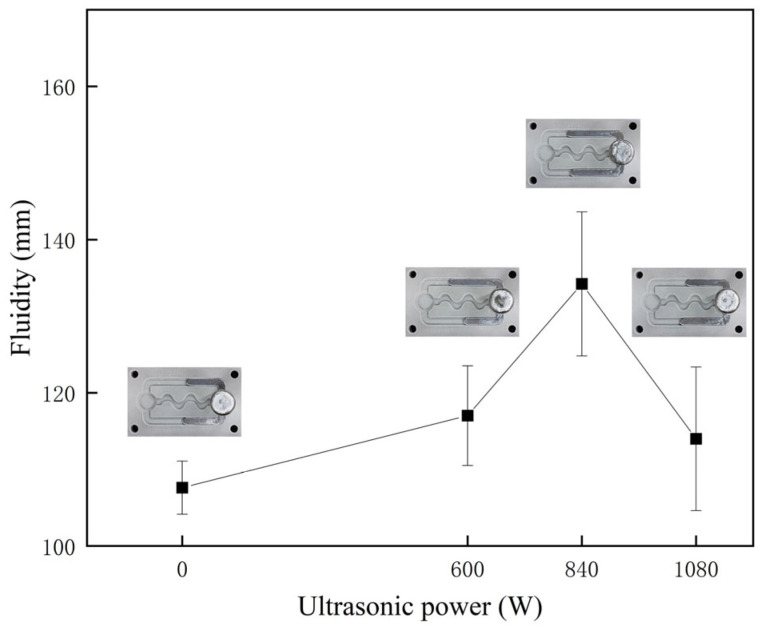
The influence of ultrasonic power on the fluidity of AlSi18 alloy.

In Figure 6, the effect of ultrasonic power on the fluidity of AlSi18 alloy is illustrated. The results indicate that as the ultrasonic power increased, the fluidity of AlSi18 alloy followed a similar trend of initially increasing and then decreasing. However, when compared to AlSi9 alloy, the influence of ultrasonic vibration on the fluidity of AlSi18 alloy was significantly weaker, as evidenced by a maximum flow length increase of only 26.2 ± 12.9 mm under 840 W ultrasonic vibration.

### 3.2. Effects of Ultrasonic Vibration on the Solidification Microstructure of Alloys

Based on the above results, it is evident that the effect of ultrasonic vibration on the fluidity of AlSi9 alloy is significantly higher than that of AlSi18 alloy, which may be attributed to their distinct solidification characteristics. To further investigate the influence of ultrasonic vibration on the solidification structure of both alloys, a comparative microstructure analysis was conducted at the same position indicated by the blue dotted box in Figure 4.

**Figure 7:**
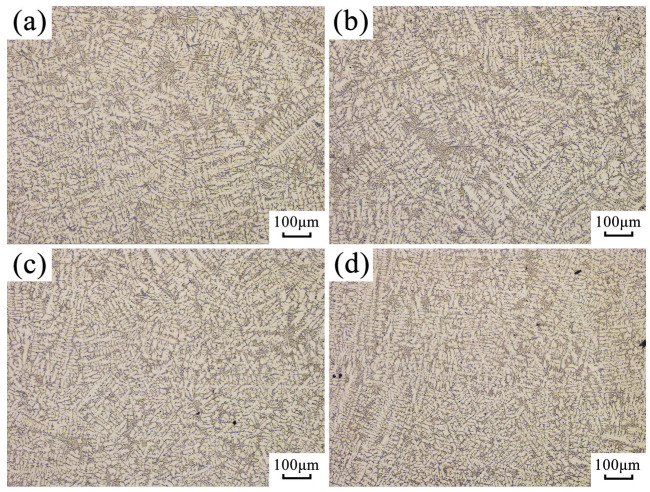
Effects of ultrasonic vibration on the solidification microstructure of hypoeutectic AlSi9 alloy: (**a**) no ultrasonic vibration; (**b**) ultrasonic power 600 W; (**c**) ultrasonic power 840 W; (**d**) ultrasonic power 1080 W.

As shown in Figure 7, the effect of ultrasonic vibration on the microstructure of hypoeutectic AlSi9 alloy was analyzed. In the absence of ultrasonic vibration, primary α (Al) exhibited a typical dendritic morphology, and coarse needle-like eutectic silicon was distributed between the dendrites. Upon the application of ultrasonic vibration, the α (Al) dendrites tended to break into short rods, and the degree of breaking was proportional to the ultrasonic power. Moreover, the secondary dendrite spacing at the sampling position decreased significantly with the increase in ultrasonic vibration power, as demonstrated in Table 3.

**Figure 8:**
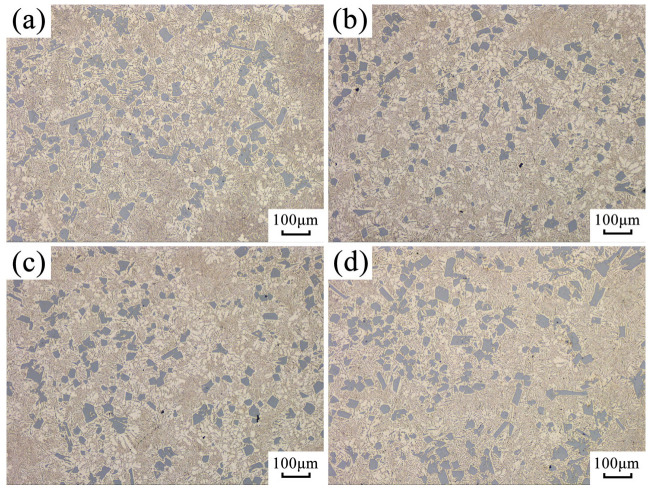
Effect of ultrasonic vibration on the solidification microstructure of hypoeutectic AlSi18 alloy: (**a**) no ultrasonic vibration; (**b**) ultrasonic power 600 W; (**c**) ultrasonic power 840 W; (**d**) ultrasonic power 1080 W.

Different from AlSi9 hypoeutectic alloy, the primary crystalline phase of hypereutectic AlSi18 alloy is the Si phase. The effect of ultrasonic vibration on its microstructure is illustrated in Figure 8, which indicates that ultrasonic vibration had no significant impact on the morphology and size of primary silicon and the eutectic structure.

**Figure 9:**
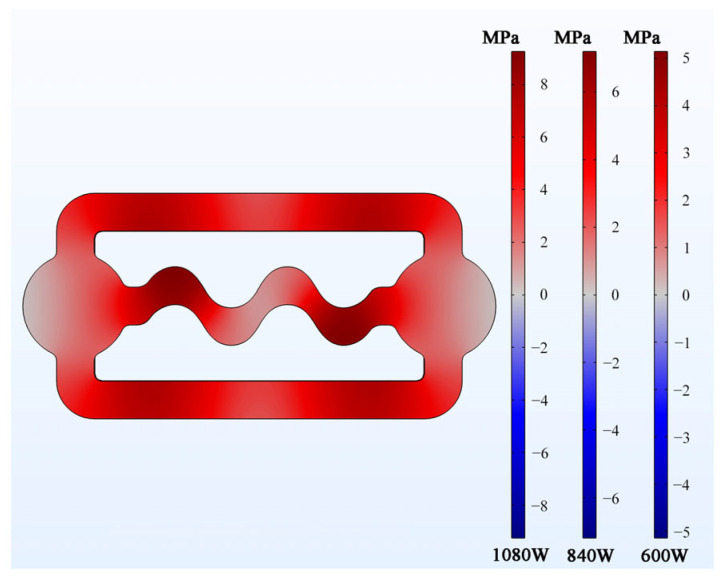
Simulated acoustic pressure (root mean square) in the melt.

Previous studies have demonstrated that ultrasonic vibration can disrupt the growth of dendrites during solidification through cavitation and acoustic flow mechanisms [23,24,26], which can ultimately enhance the fluidity of the alloy [25]. In this regard, the acoustic pressure distribution in the alloy melt was calculated, assuming that the cavity was completely filled with melt without any solidification. The density and sound velocity of liquid alloy at a temperature of 973 K were 2.35 × 10^3^ kg·m^−3^ and 5.496 × 10^3^ m·s^−1^, respectively. Figure 9 illustrates that the maximum acoustic pressure in the melt ranged from 3–8 MPa at different input powers, which exceeds the reported cavitation threshold of 1 MPa in aluminum alloy [1]. It is noteworthy that all the ultrasonic power levels used in this experiment could theoretically generate cavitation effects in the melt near the mold cavity wall. However, during solidification, when the shell begins to form, the actual ultrasonic power propagates to the melt adjacent to the solidification front, and it will subsequently weaken. Therefore, it is necessary to further investigate whether cavitation can occur in the actual melt during solidification.

As discussed above in Figure 7, the dendrites of AlSi9 alloy were effectively broken by ultrasonic vibration, consequently helping to improve the fluidity of the alloy. The dendrites here may be broken by ultrasonic cavitation or ultrasonic mechanical vibration.

It has been proven that high-energy ultrasonic vibration can promote the heterogeneous nucleation of primary silicon and effectively refine the primary silicon of AlSi18 alloy [27]. However, in the present work, the size and morphology of primary silicon in AlSi18 alloy shown in Figure 8 were not influenced by ultrasonic vibration. This may indirectly verify that due to the high solidification rate and fast formation of the solidified shell, ultrasonic vibration attenuates when it passes through the interface between the solidified shell and the mold. Therefore, the ultrasonic intensity transmitted to the melt at the solidification front is not enough to produce a cavitation effect, and the solidification front is then only affected by the mechanical ultrasonic vibration and acoustic flow effect. Under this condition, the effect of ultrasonic cavitation promoting the heterogeneous nucleation of primary silicon by improving the wettability of nucleation particles [28] has not occurred here. In addition, the crystallization of primary silicon is in the form of non-dendritic and faceted growth, so the mechanical vibration and acoustic flow caused by ultrasonic vibration also cannot break the growing primary silicon. Therefore, the effect of ultrasonic vibration on refining grain by promoting heterogeneous nucleation and broken dendrites is not reflected in hypereutectic AlSi18 alloy. However, even in the absence of cavitation phenomena within the melt, forced convection generated by ultrasonication can fragment the coarse dendritic grains [5]. The hypoeutectic AlSi9 alloy used in this study also has well-developed α (Al) dendritic structures, and its dendritic structure was significantly refined under the action of forced convection (as shown in Figure 8). For AlSi9 alloy with a well-developed α (Al) dendritic structure, the continuously growing dendrites during solidification will form a net-like structure, thereby increasing flow resistance inside the melt. When subjected to ultrasonic vibration of the mold, the well-developed dendritic structure inside the melt is destroyed, reducing the flow resistance of the mushy zone, which is very beneficial for promoting the fluidity of the melt. Since hypereutectic AlSi18 alloy does not have an obvious dendritic structure during solidification, the ultrasonic vibration of the mold cannot reduce the flow resistance inside its melt by destroying dendrites, thereby improving the fluidity of the alloy. Therefore, in this study, the promoting effect of mold ultrasonic vibration on the fluidity of AlSi9 alloy was much better than that of AlSi18 alloy.

**Figure 10:**
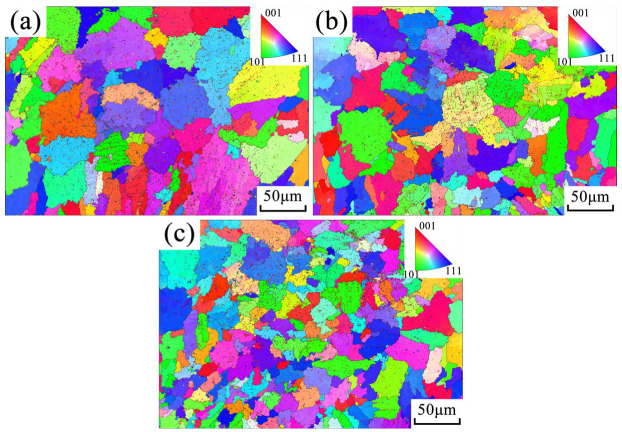
EBSD maps of AlSi9 alloy obtained under different power ultrasonic vibrations: (**a**) 0 W; (**b**) 840 W; (**c**) 1080 W.

**Figure 11:**
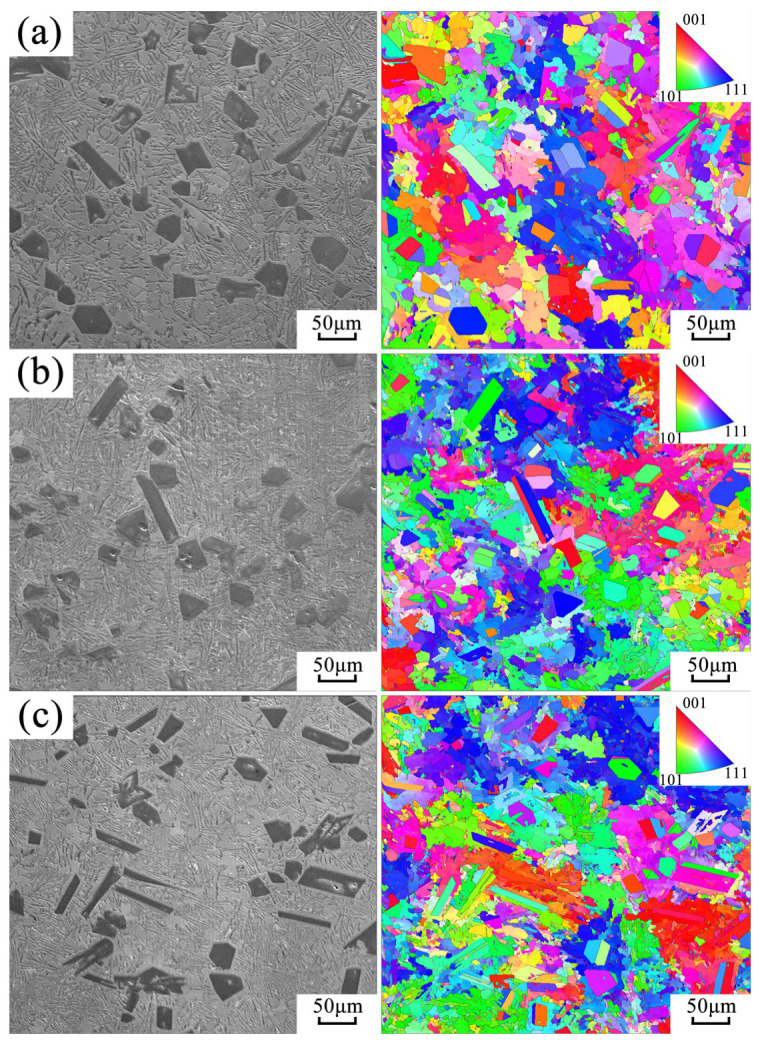
EBSD maps of AlSi18 alloy obtained under different power ultrasonic vibrations: (**a**) 0 W; (**b**) 840 W; (**c**) 1080 W.

The grain size and distribution of both AlSi9 and AlSi18 alloys were analyzed further using electron backscatter diffraction (EBSD), as presented in Figure 10 and Figure 11. As shown in Figure 10, ultrasonic vibration significantly reduced the average grain size of AlSi9 alloy. With the increase in ultrasonic power, the average grain size decreased significantly, and the grain refinement effect was found to be proportional to the ultrasonic input power, as summarized in Table 4. Therefore, this indicates that ultrasonic vibration-induced fragmentation of the primary α (Al) dendrites of AlSi9 alloy not only enhances its fluidity but also refines the grain size through dendrite detachment and multiplication. However, for the hypereutectic AlSi18 alloy shown in Figure 11, both the primary Si and eutectic structure were not significantly refined by ultrasonic vibration, consistent with the above deduction.

As discussed above, ultrasonic vibration can effectively enhance the fluidity of the melt by breaking dendrites, especially for hypoeutectic AlSi9 alloy. However, the extent of dendrite fragmentation was proportional to the ultrasonic power (0–1080 W), and the fluidity of the alloy did not improve further at high power (1080 W) but instead decreased. Furthermore, although ultrasonic vibration did not change the solidification and corresponding structure of AlSi18, it did have an impact on its fluidity to some extent. Therefore, it is speculated that ultrasonic vibration may not only affect fluidity by interfering with the solidification process but also through the hydrodynamic aspects of melt flow, which will be discussed below.

### 3.3. Effect of Ultrasonic Vibration on the Hydrodynamics of Melt Flow

**Figure 12:**
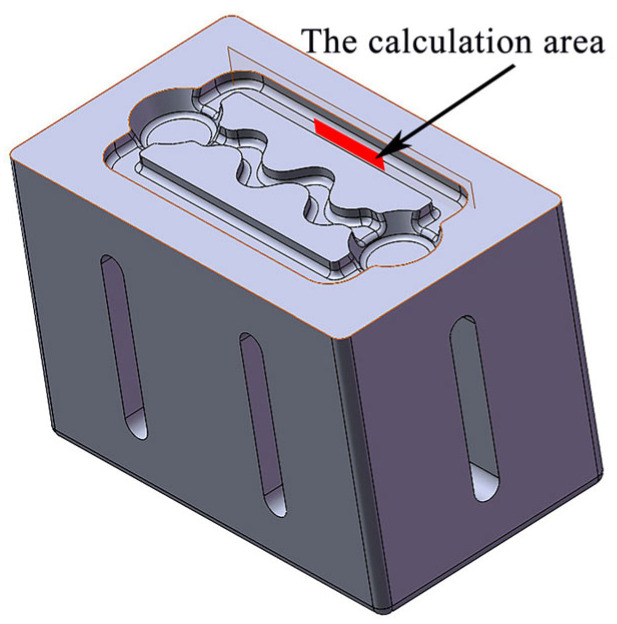
Calculation area for ANSYS-FLUENT.

**Figure 13:**
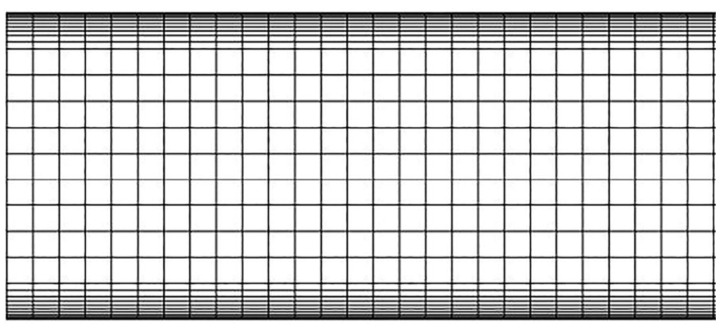
Schematic diagram of boundary layer mesh.

“ANSYS-FLUENT” was used to examine the effect of ultrasonic vibration on the flow field of aluminum alloy melt. The middle section of the straight channel of the mold cavity was extracted as the calculation area (long 50 mm × high 5 mm), as shown in Figure 12. To obtain accuracy of the computational results, it was necessary to generate boundary layer mesh during the process of meshing the model, as illustrated in Figure 13. The near-wall treatment adopted enhanced wall function. Solver selected the pressure solver. The SIMPLE algorithm was used for pressure and velocity coupling.

To apply ultrasonic vibration, a user-defined function (UDF) was used to define the vibration displacement and velocity boundary conditions on the bottom of the calculated region. The bottom wall was made to vibrate perpendicularly to the axis direction (x-axis direction) of the straight passage, and the harmonic vibration speed rule was defined using Formula (1). The value of vibration amplitude *A* was chosen based on the displacement value (ranging from 0 to 25 μm) in Figure 4, which depends on the ultrasonic power used. The time step was determined by the vibration period (*T*) of the wall: *T*/16.

v = 2π*fA*cos(2π*ft*), (1)

where *f* is frequency (24,000 Hz), *t* is time, and *A* is only related to amplitude.

The flow models used for the simulation calculation depended on the flow conditions, which could be influenced by ultrasonic vibration. Thus, in the simulation process, the laminar flow model was first used to simulate fluid flow. The flow rate of the fluid was obtained from the simulation results, and then the Reynolds number of the fluid was calculated using Formula (2). When the Reynolds number of the fluid exceeded 2300, the k-ε turbulence model was then used to simulate the fluid.

Re = *ρvd*/*μ*, (2)

where *ρ* is the density (10^3^ kg·m^−3^), *v* is the flow rate, *d* is the characteristic length (4.55 × 10^−3^ m), and *μ* is the viscosity coefficient (1.01 × 10^−3^ Pa·s).

After measurement, the flow velocity of the molten metal in the runner during casting was determined to be approximately 1.5 m·s^−1^, and this velocity was used as the initial velocity for the fluid simulation. As shown in Table 5, the Reynolds coefficient of the fluid increased with the vibration amplitude of the wall, indicating an increasing disturbance in the flow field. When the wall vibration amplitude reached 20 μm, the Reynolds number exceeded 2300, and turbulence occurred.

**Figure 14:**
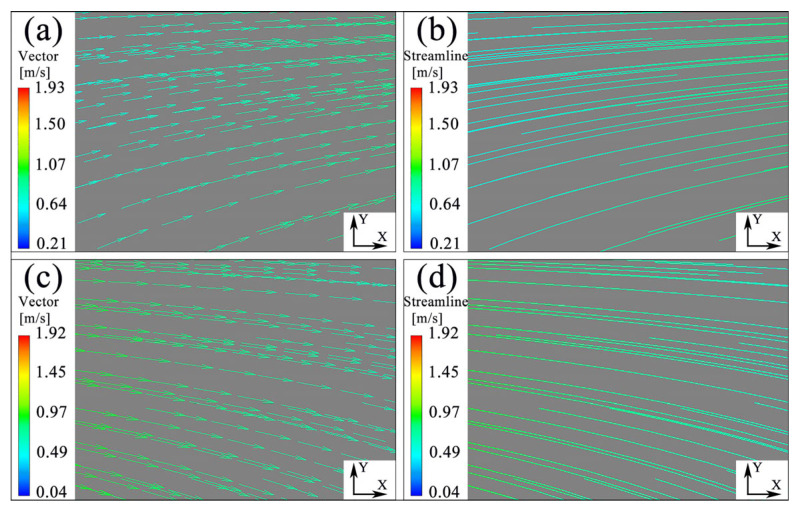
Velocity and streamline diagram of fluid at π/2 and 3π/2 phase: (**a**) velocity diagram of fluid at π/2 phase; (**b**) streamline diagram of fluid at π/2 phase; (**c**) velocity diagram of fluid at 3π/2 phase; (**d**) streamline diagram of fluid at 3π/2 phase.

**Figure 15:**
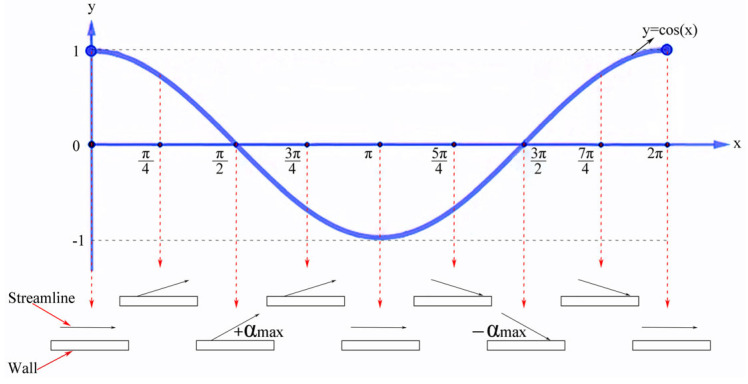
Phase-dependent variation of the angle between streamline and wall.

**Figure 16:**
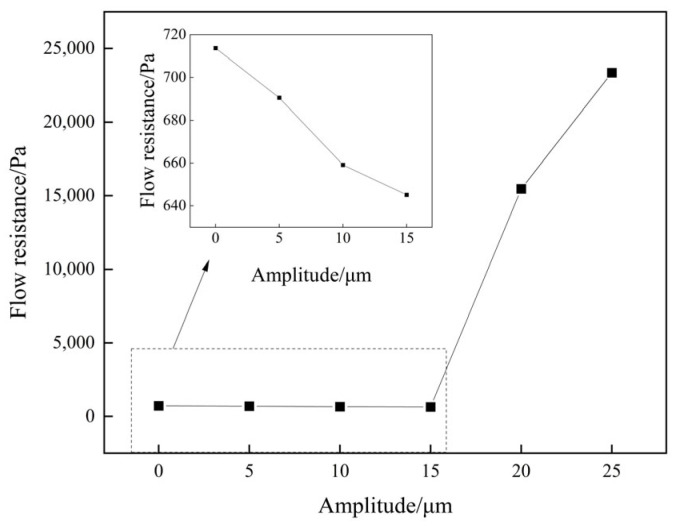
Variation of periodic average flow resistance with frequency under different amplitudes.

Figure 14 illustrates the relative velocity vector and streamline diagrams of the flow field obtained under a wall vibration amplitude of 15 μm. The results show that the upward and downward ultrasonic vibrations of the wall induce diagonal fluid flows, with periodic action occurring in one ultrasonic vibration cycle. A schematic description of the periodic action of the fluid is provided in Figure 15. During wall vibration, the angle (α) between the streamline and wall varies from −αmax to +αmax with the change in vibration phase position. Then, +α_max_ and −α_max_ are the two extremes corresponding to the π/2 and 3π/2 phase positions, respectively. The periodic upward and downward flow diversion of the fluid could influence wall shear stress. In one vibration cycle, the wall shear stress increases, with α varying from 0 to +α_max_, and decreases, with α varying from 0 to −α_max_. The impact of periodically varying wall shear stress on the fluid can be measured by its flow resistance. Figure 16 displays the average flow resistance of fluid over 30 cycles under ultrasonic vibrations of different amplitudes. It was observed that the flow resistance decreased with ultrasonic vibration amplitudes of less than 15 μm. However, when the ultrasonic vibration intensity reached a certain level (amplitude > 20 μm or input ultrasonic power > 1080 W), turbulence occurred in the fluid, greatly increasing the flow resistance and leading to a loss of flowing kinetic energy.

In the flow process of AlSi9 and AlSi18 alloys under ultrasonic vibration, only from the hydrodynamics aspect, the melt flow resistance decreased with the increase in ultrasonic vibration power from 0 to 840 W, especially for AlSi18 alloy, whose fluidity under ultrasonic vibration was almost irrelevant to solidification behavior. However, when the ultrasonic power reaches 1080 W (amplitude > 20 μm), turbulence will occur in the melt, resulting in a serious loss of the flowing kinetic energy of the melt. As discussed above, the fluidity of AlSi9 alloy was improved by ultrasonic vibration not only from the hydrodynamics aspect but also greatly from the solidification aspect (breaking the growing dendrites in the mushy zone). However, the flowing energy loss caused by turbulence under 1080 W ultrasonic vibration (amplitude > 20 μm) greatly counteracted the promoting effect on the fluidity by breaking dendrites.

It can be found that ultrasonic vibration can affect the fluidity of alloys from the two aspects of hydrodynamics and dendrite growth. For the alloy without obvious dendrite growth characteristics (AlSi18 alloy), ultrasonic vibration affected the fluidity of the alloy mainly from the hydrodynamics aspects: ultrasonic vibration can reduce the flow resistance of melt to improve fluidity. However, high-intensity vibration (amplitude > 20 μm, ultrasonic power > 1080 W) could induce turbulence in the melt, which is unfavorable for fluidity. For the alloy with typical dendrite growth in solidification (AlSi9 alloy), it can improve the fluidity of the alloy by hydrodynamically reducing the flow resistance of the melt and breaking the growing dendrites in the mushy zone, with the latter aspect being more prominent. However, the energy loss caused by the turbulence under 1080 W ultrasonic vibration (amplitude > 20 μm) largely counteracted the promoting effect of interrupting dendrites on fluidity.

## 4. Conclusions

This study investigated the effects of ultrasonic vibration on the solidification microstructure and fluidity of cast aluminum alloys (AlSi9 and AlSi18) with different solidification characteristics using experimental and simulation analyses. The results show that the grain size of AlSi9 alloy, with its obvious dendritic structure, was refined under the action of ultrasonic vibration, while there was no significant change in the solidification structure of AlSi18 alloy, which does not have an obvious dendritic structure. With the increase in ultrasonic power, the fluidity of both alloys showed a trend of first increasing and then decreasing, with the effect of ultrasonic vibration on the fluidity of hypoeutectic AlSi9 alloy being more significant.

It can be found that ultrasonic vibration can affect the fluidity of alloys in both solidification and hydrodynamics aspects. For AlSi18 alloy, which lacks dendrite growing solidification characteristics, the microstructure is not significantly influenced by ultrasonic vibration, and its fluidity is mainly affected by the hydrodynamics aspect. Specifically, appropriate ultrasonic vibration can reduce the flow resistance of the melt and improve fluidity. However, when the vibration intensity is high enough to induce turbulence in the melt, the turbulence will greatly increase flow resistance and decrease fluidity. On the other hand, for AlSi9 alloy, with its obvious dendrite growing solidification characteristics, ultrasonic vibration can affect solidification by breaking the growing α (Al) dendrites, leading to refinement of the solidification microstructure. Ultrasonic vibration can then improve the fluidity of AlSi9 alloy not only from the hydrodynamics aspect but also by breaking the dendrite network in the mushy zone to decrease flow resistance, with the latter aspect being more prominent. However, the energy loss caused by turbulence under high-intensity ultrasonic vibration can largely counteract the promoting effect of breaking dendrites on fluidity.

## Figures and Tables

**Table 1:** Material properties used in numerical simulation.

Materials	Density (kg·m^−3^)	Young’s Modulus (GPa)	Poisson’s Ratio
Structural steel	7850	200	0.30
Aluminum	2700	70	0.33
Ti-6Al-4V	4510	113	0.34
H13	7000	140	0.30

**Table 2:** Schemes for the application of ultrasonic vibration.

Schemes	Alloy	Solidification Temperature Range (K)	Pouring Temperature (K)	Theoretical Degree of Superheat (K)	Ultrasonic Power (W)
1	AlSi9	865–807	973	108	0
2					600
3					840
4					1080
5	AlSi18	934–850	1003	69	0
6					600
7					840
8					1080

**Table 3:** Influence of ultrasonic vibration on secondary dendrite spacing of AlSi9 alloy.

Ultrasonic Power (W)	Average Secondary Dendrite Spacing (μm)
0	12.45 ± 0.33
600	9.64 ± 0.34
840	8.42 ± 0.17
1080	7.55 ± 0.34

**Table 4:** Average grain size (μm) of AlSi9 alloy.

Ultrasonic Power (W)	Average Grain Size (μm)
0	352 ± 23
840	214 ± 37
1080	153 ± 16

**Table 5:** Reynolds number of fluids in different ultrasonic vibration amplitude simulations.

Amplitude (μm)	Reynolds Number
0	284
5	314
10	338
15	362
20	2634
25	3246

## Data Availability

The data presented in this study are available from the corresponding author upon reasonable request.
